# Effect of Light/Dark Cycle on Photosynthetic Pathway Switching and CO_2_ Absorption in Two *Dendrobium* Species

**DOI:** 10.3389/fpls.2019.00659

**Published:** 2019-05-22

**Authors:** Yongsan Cheng, Dongxian He, Jie He, Genhua Niu, Rongfu Gao

**Affiliations:** ^1^ Key Laboratory Agricultural Engineering in Structure and Environment, Ministry of Agriculture and Rural Affairs, China Agricultural University, Beijing, China; ^2^ National Institute of Education, Nanyang Technological University, Singapore, Singapore; ^3^ Texas A&M AgriLife Research at El Paso, Texas A&M University System, El Paso, TX, United States; ^4^ College of Biological Sciences and Technology, Beijing Forestry University, Beijing, China

**Keywords:** C3-like pathway, CAM pathway, *Dendrobium officinale*, *D. primulinum*, dark net CO_2_ exchange percentage, stomatal behavior

## Abstract

Many *Dendrobium* species are both ornamental and medicinal plants in China. Several wild species have been exploited to near extinction, and facility cultivation has become an important way to meet the great market demand. Most *Dendrobium* species have evolved into crassulacean acid metabolism (CAM) pathways in adapting to harsh epiphytic environment, leading to low daily net CO_2_ absorption. Photosynthetic pathways of many facultative CAM plants are regulated by various environmental factors. Light/dark cycle plays an important role in regulating the photosynthetic pathway of several CAM species. The aims of this study were to investigate whether the photosynthetic pathway of *Dendrobium* species could be regulated between C3 and CAM by changing light/dark cycles and the daily net CO_2_ absorption could be enhanced by shortening light/dark cycle. In this study, net CO_2_ exchange rates of *D. officinale* and *D. primulinum* were monitored continuously during two different light/dark cycles conversion compared to *Kalanchoe daigremontiana* as an obligate CAM plant. The net CO_2_ exchange pattern and stomatal behavior of *D. officinale* and *D. primulinum* were switched from CAM to C3-like by changing the light/dark cycle from 12/12 h to 4/4 h. However, this switching was not completely reversible. Compared to the original 12/12 h light/dark cycle, the dark, light, and daily net CO_2_ exchange amount of *D. officinale* were significantly increased after the light/dark cycle was changed from 4/4 h to 12/12 h, but those in *D. primulinum* was opposite and those in *K. daigremontiana* was not affected. Daily net CO_2_ exchange amount of *D. officinale* increased by 47% after the light/dark cycle was changed from 12/12 h to 4/4 h, due to the sharp increase of light net CO_2_ exchange amount. However, the large decrease of dark net CO_2_ exchange amount could not be offset by increased light net CO_2_ exchange amount, leading to reduced daily net CO_2_ exchange amount of *D. primulinum*. In conclusion, the 4/4 h light/dark cycle can induce the photosynthetic pathway of *D. officinale* and *D. primulinum* to C3-like, and improve the daily CO_2_ absorption of *D. officinale*.

## Introduction

*Dendrobium* is the second largest genus of Orchidaceae (Takamiya et al., 2011; Yan et al., 2015). Many *Dendrobium* species not only have important ornamental values but also possess high medicinal values in China (Ng et al., 2012; Yan et al., 2015; Teixeira Da Silva et al., 2016). Many wild species are endangered due to the exploitation, and facility cultivation has become an important way to meet the great market demand of *Dendrobium*. However, most *Dendrobium* species have evolved into crassulacean acid metabolism (CAM) pathways in adapting to harsh epiphytic environment, and their photosynthetic pathways also vary with the environment and species (Gehrig et al., 2001; Su and Zhang, 2003; Yang et al., 2011; Ren and Bai, 2015). For instance, Su and Zhang (2003) measured the daily changes of net CO_2_ exchange rates of *D. officinale* under various weather conditions and found that it had a CAM pattern on sunny days, a C3 pattern on rainy days, and a pattern between CAM and C3 on cloudy days. Yang reported that diurnal net CO_2_ exchange rates in *D. primulinum* had four distinct phases of 24-h CAM cycle (Yang et al., 2011). More and more *Dendrobium* plants have been found to have CAM pathway to some extent.

It is widely assumed that the very earliest evolution of CAM was driven by low ambient atmospheric CO_2_, then the requirement of economic water use for terrestrial CAM plants (Flexas et al., 2012). CAM is found in about 6% of vascular plants species spanning 35 plant families as an adaption to water deficit (Silvera et al., 2010). CAM is an important ecophysiological metabolic adaption that permits plants to occupy extremely arid environments (e.g., deserts), semi-arid regions with seasonal water availability (e.g., Mediterranean climates), or habitats with intermittent water supply (e.g., tropical epiphytic habitats) (Cushman, 2001). CAM is often described as a continuum, with constitute CAM at one end, C3 at the other, and various facultative CAM between (Winter et al., 2015; Winter, 2019). Constitutive CAM species undergo a one-way developmental progression to CAM and maintain CAM even under well-watered conditions (Winter et al., 2008). Facultative CAM describes the optional use of CAM photosynthesis in plants that otherwise employ C3 or C4 photosynthesis; reversibility distinguishes facultative CAM from ontogenetically programmed unidirectional C3-to-CAM shifts inherent in constitutive CAM plants (Winter and Holtum, 2014).

Photosynthetic pathways of many facultative CAM species are influenced by several environmental factors such as soil water content and light intensity, as well as photoperiod (Brulfert et al., 1996; Mattos and Lüttge, 2001; Brilhaus et al., 2016;). CAM and C3-like pathway of *Kalanchoe blossfeldiana* can be induced by short light period and short light period with interruption of long dark period respectively, and phytochrome is involved in these processes (Schmitz, 1951; Gregory and Thimann, 1954; Wilkins, 1962; Queiroz and Morel, 1974). Studies on the effects of *D. ekapol* showed that short light period increased the net CO_2_ absorption of phase I (dark period), while long light period increased the net CO_2_ absorption of phase II (early stage of light period) and phase IV (at the end of light period) (Sekizuka et al., 1995). The CAM pathway and CO_2_ uptake of *Doritaenopsis* Queen Beer “Mantefon” can both be restrained by short light/dark cycle (Kim et al., 2017). Under the normal 12/12 h light/dark cycle, CAM and C3 pathways coexist in *D. officinale*, whereas C3 pathway can be upregulated by short light/dark cycle (short light period and short dark period), especially extreme treatment of 4/4 h light/dark cycle can lead to C3-like light-only CO_2_ uptake pattern (Zhang et al., 2014). However, it was not clear whether stomatal movement was involved or whether photosynthetic pathway switching was reversible between light/dark cycles of 12/12 h and 4/4 h.

For a long time, both C3 and CAM stomatal movements have been associated with the perception of CO_2_ concentration; intuitively, the response to partial pressure of CO_2_ in the substomatal cavities (Ci) seems to be the most likely signal to regulate the inverse stomatal cycle associated with CAM (Males and Griffiths, 2017). At the beginning of phase I of CAM, stomatal opening was considered to be caused by the decrease of Ci with the increase of phosphoenolpyruvate carboxylase (PEPC) activity at dusk (Griffiths et al., 2007; Caemmerer and Griffiths, 2009). In the morning, stored malic acid is decarboxylated in phase II, which strengthens stomatal closure. This, coupled with respiration, can lead to 100 times atmospheric concentration in Ci. The phase IV of reopening stomata is related to the end of malic acid decomposition, therefore, internal CO_2_ limitation (Cockburn, 1979). When CO_2_ uptake and malic acid accumulation decreased at night and subsequent Ci regeneration decreased at phase III, stomata remained closed, and there was almost no transient response to CO_2_, suggesting that circadian control of stomata was still a key factor in controlling CAM cycles of *Kalanchoe daigremontiana* and *K. pinnata* (Caemmerer and Griffiths, 2009). Studies on facultative CAM plants show that blue light can regulate stomatal conductance opening only in C3 mode (Lee and Assmann, 1992; Tallman et al., 1997).

This study aimed to investigate whether photosynthetic pathway of *Dendrobium* plants could be switched between CAM and C3, and the daily net CO_2_ absorption could be increased by different light/dark cycles conversion. The results of this study may help researchers better understand the relationship between light/dark cycle, stomatal behavior, and CO_2_ absorption in different CAM plants.

## Materials and Methods

### Experimental Materials and Cultivation Methods

*Dendrobium officinale* collected from Jinhua city (Zhejiang, China) and *D. primulinum* collected from Puer city (Yunnan, China) were cultivated and acclimatized in a walk-in phytotron at China Agricultural University (Beijing, China) for 2 years. *K. daigremontiana*, an obligate CAM plant, used as a reference, was grown from leaf-borne ramets in the same walk-in phytotron. The two *Dendrobium* species and *K. daigremontiana* were planted in 0.4 L plastic pots, three plants per pot. The cultivation substrate was moss for these two *Dendrobium* species, and a mixture of vermiculite and perlite (volume ratio was 3:1) for *K. daigremontiana*. All pots were irrigated with 70 ml tap water every 2 days, and irrigated with the same amount of nutrient solution every 7 days for 2 years. Nutrient solution formula was as follows: Ca(NO_3_)_2_•4H_2_O 205 mg L^−1^, MgSO_4_•7H_2_O 60 mg L^−1^, KH_2_PO_4_ 136 mg L^−1^, NH_4_NO_3_ 80 mg L^−1^, MnSO_4_•H_2_O 3.6 mg L^−1^, H_3_BO_3_ 2.7 mg L^−1^, FeSO_4_•7H_2_O 13.4 mg L^−1^, CuSO_4_•5H_2_O 0.1 mg L^−1^, ZnSO_4_•7H_2_O 0.4 mg L^−1^, and (NH_4_)_6_Mo_7_O_24_•4H_2_O 0.1 mg L^−1^. EC and pH of the nutrient solution were controlled at 0.6–0.7 ms cm^−1^ and 6.0–6.5, respectively. The environmental parameters of the phytotron for 2 years of long-term cultivation were as follows: artificial light source was T5 tricolor fluorescent lamp (28 W, Beijing Lighting Valley Co., Ltd., Beijing, China); the photosynthetic photon flux density (PPFD) at the plant canopy was 150 μmol m^−2^ s^−1^; light/dark cycle was 12/12 h (light period, 0800–2000 hours; dark period, 2000–0800 hours); temperature during the light and dark periods was 26°C ± 1 and 22°C ± 1°C, respectively; relative humidity was 65% ± 5%; CO_2_ concentration was 500 ± 50 μmol mol^−1^.

### Light/Dark Cycle Treatment

Different light/dark cycle treatments began after acclimatization in the environment of phytotron for 3 months, when both *D. officinale* and *D. primulinum* plants had 8–10 expanded leaves, and *K. daigremontiana* plants had 16 expanded leaves. All the plants used in this experiment were in a vegetative stage when they were subjected to different light/dark cycle treatments. Twelve similar-sized healthy plants were selected from each species treating with a light/dark cycle of 12/12 h (light period, 0800–2000 hours; dark period, 2000–0800 hours) for five cycles (5 days). At the end of the fifth dark period, the light/dark cycle was changed to 4/4 h for 15 cycles (5 days). After the 15th dark period (the end of the fifth day), the light/dark cycle was changed back to 12/12 h for another five cycles (5 days) as mentioned above. The light intensity, temperature, relative humidity, and CO_2_ concentration during the light/dark cycle treatment were maintained at the same levels as mentioned above.

### Measurement of Net CO_2_ Exchange Rates, Dark, Light, and Daily Net CO_2_ Exchange Amount, and Dark Net CO_2_ Exchange Percentage

The photosynthetic continuous measurement system (Zhang et al., 2014) used in this study consists of four cuvettes (25 cm × 15 cm × 6 cm), a host computer, and an IRGA CO_2_ analyzer (LI-7000, LICOR, Lincoln, USA). One shoot of *D. officinale* and one shoot of *D. primulinum*, each with 8–10 leaves, as well as two fully expanded mature leaves from two different *K. daigremontiana* plants were selected for this measurement. Each of the four different samples was enclosed into each of the different cuvettes. All leaves were held flat by several horizontal nylon wires. All plants were kept intact and irrigated with 70 ml tap water once a day during the measurement. The measurement was repeated for three times. The temperature and relative humidity of the cuvettes was the same level as that of the phytotron as mentioned above. PPFD at the top of the cuvettes was 150 μmol m^−2^ s^−1^. The air flow rate of each cuvette was 1.0 L min^−1^. The difference of CO_2_ concentration between reference and sample gas of each cuvette recorded every 10 min throughout the treatment period. The measurements for these three species were carried out concurrently. Leaf area of each cuvette was determined according to Yang et al. (2002) after 15 days. Then net CO_2_ exchange rate of each cuvette was calculated according to Zhang et al. (2014). Dark net CO_2_ exchange amount, light net CO_2_ exchange amount, and daily net CO_2_ exchange amount were integrated based on dark, light, and daily net CO_2_ exchange rates everyday (24 h). The dark net CO_2_ exchange percentage was defined as dark net CO_2_ exchange amount divided by daily net CO_2_ exchange amount times 100%. Dark, light, and daily net CO_2_ exchange amount, as well as dark net CO_2_ exchange percentage measurement data for these three species were collected at the last 2 days of each light/dark cycle (days 4 and 5, days 9 and 10, days 14 and 15). Three replicates were conducted in this measurement for each species.

### Measurement of Stomatal Conductance

A leaf porometer (SC-1, Decagon, Washington, USA) was used to measure the stomatal conductance of abaxial surface of the leaves of other plants outside the cuvettes for *D. officinale*, *D. primulinum*, and *K. daigremontiana*. Daily desiccant replacement and the leaf porometer calibration were done before the measurement. Automatic mode was used to obtain each value in 30 s. Measurements were conducted at 0700, 0900, 1,300, 1,600, 1900, 2100, and 2300 hours on day 5 (0700, 2100, and 2300 hours were in dark period), and at 0200, 0600, 1000, 1400, 1800, and 2,200 hours on day 9 (0600, 1400, and 2200 hours were in dark period). Four plants per species were measured at each time. The stomatal conductance were measured for the third leaf from top of each plant (*n* = 4).

### Statistical Analysis

Statistics analysis was performed using the IBM SPSS Statistics 21 (IBM, Inc., Armonk, NY, USA). The average dark, light, and daily net CO_2_ exchange amount, as well as dark net CO_2_ exchange percentage for each species (*D. officinale*, *D. primulinum*, and *K. daigremontiana*) were compared respectively between different light/dark cycles by Duncan’s multiple range test at *p* < 0.05. The average stomatal conductances at each time of day 5 and day 9 were compared for each species by the same method as mentioned above, respectively.

## Results

### Net CO_2_ Exchange Rates

**Figure 1:**
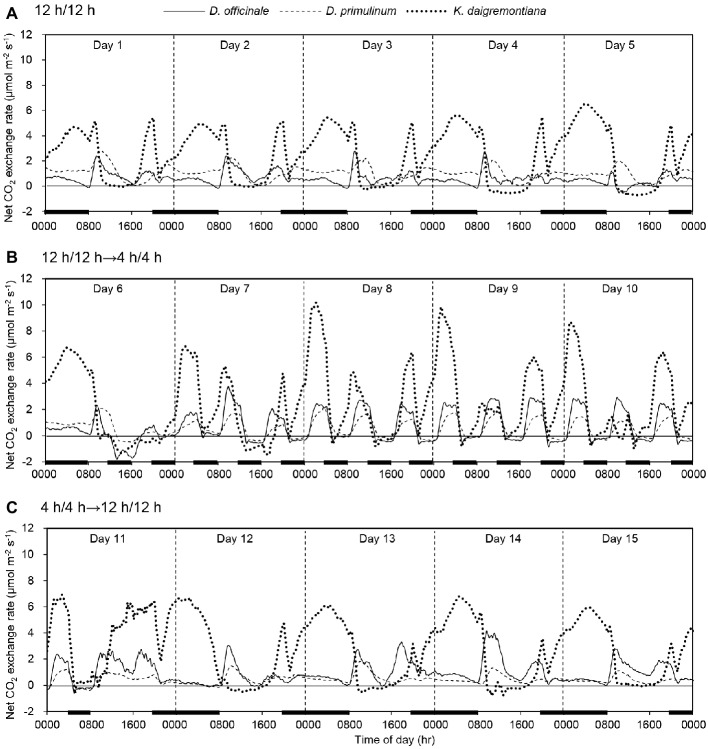
Effect of light/dark cycle on net CO_2_ exchange rates of *D. officinale*, *D. primulinum*, and *K. daigremontiana*. Light/dark cycle was 12/12 h from day 1 to day 5 **(A)**, 4/4 h from day 6 to day 10 **(B)**, and 12/12 h from day 11 to day 15 **(C)**. The thin black line on the horizontal axis indicates light period, and the thick black line indicates dark period.

The net CO_2_ exchange exhibited obvious trailing phenomenon during the conversion between the light and dark period. Thus its influence had been considered in the subsequent results analysis. When light/dark cycle was 12/12 h, net CO_2_ exchange rates of *D. officinale*, *D. primulinum*, and *K. daigremontiana* exhibited multiple periodic variations. The net CO_2_ exchange rates of *D. officinale*, *D. primulinum*, and *K. daigremontiana* increased successively after onset of the dark period. When switched to light period, the net CO_2_ exchange rate of *K. daigremontiana* first rose to the peak and then fell to near zero at 0900 hours; that of *D. officinale* and *D. primulinum* first increased and then decreased until 1600 hours before falling to near zero. The net CO_2_ exchange rates of *D. officinale* exhibited the shortest time of fluctuation around zero, followed by *D. primulinum*, that of *K. daigremontiana* exhibited the longest duration near zero. The net CO_2_ exchange rates of all these three species would rise at the end of the light period (Figure 1A). After light/dark cycle was changed to 4/4 h for 3 days (on day 9), net CO_2_ exchange of *D. officinale* and *D. primulinum* showed C3-like pattern, with net CO_2_ uptake in light period and net CO_2_ release in dark period, whereas net CO_2_ uptake in dark period maintained in *K. daigremontiana*. Net CO_2_ exchange rate of *K. daigremontiana* at 0200 hours was higher than that at 1000 and 1800 hours (Figure 1B). After the light/dark cycle was changed back to 12/12 h for 1 day, net CO_2_ exchange of these three species also switched back to the similar pattern of earlier light/dark cycle of 12/12 h (Figure 1C).

### Dark, Light, and Daily Net CO_2_ Exchange Amount

**Figure 2:**
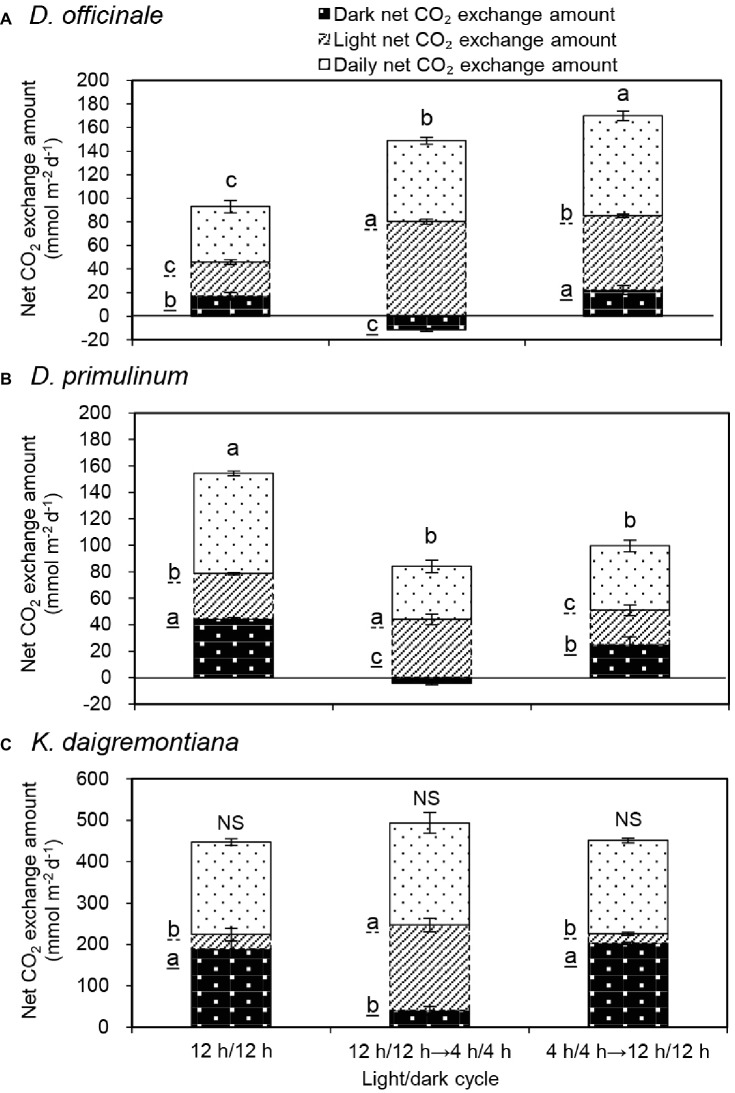
Effect of light/dark cycle on dark net CO_2_ exchange amount, light net CO_2_ exchange amount, and daily net CO_2_ exchange amount of *D. officinale* **(A)**, *D. primulinum* **(B)**, and *K. daigremontiana* **(C)**. Daily net CO_2_ exchange amount is the sum of dark net CO_2_ exchange amount and light net CO_2_ exchange amount. Each point is the mean of three measurements of three different plants. Vertical bars indicate the standard deviations. Different letters indicate statistically significant differences by Duncan’s multiple range test (*p* < 0.05). The data of the last 2 days of each light/dark cycle of three repeated experiments were taken to average. The letters underlined by dotted line and solid line represent differences in the light net CO_2_ exchange amount and dark net CO_2_ exchange amount respectively.

For *D. officinale*, daily net CO_2_ exchange amount increased significantly from 47 to 69 mmol m^−2^ day^−1^ and then further increased significantly to 85 mmol m^−2^ day^−1^ when the light/dark cycle was changed from 12/12 h to 4/4 h and then back to 12/12 h (Figure 2A). After the light/dark cycle was changed from 12/12 h to 4/4 h, the increase of daily net CO_2_ exchange amount mainly resulted from the increase of light net CO_2_ exchange amount. Compared to 4/4 h light/dark cycle, daily net CO_2_ exchange amount significantly increased after light/dark cycle was changed to 12/12 h, due to the increase of dark net CO_2_ exchange amount was more than the decrease of light net CO_2_ exchange amount. Compared to the previous 12/12 h light/dark cycle, daily net CO_2_ exchange amount significantly increased after light/dark cycle was changed back to 12/12 h, due to both the increase of light net CO_2_ exchange amount and dark net CO_2_ exchange amount.

For *D. primulinum*, daily net CO_2_ exchange amount decreased significantly from 76 to 40 mmol m^−2^ day^−1^ and then remained no significantly changed when the light/dark cycle was changed from 12/12 h to 4/4 h then back to 12/12 h (Figure 2B). After the light/dark cycle was changed from 12/12 h to 4/4 h, the decrease in daily net CO_2_ exchange amount mainly resulted from the large decrease of dark net CO_2_ exchange amount, which offset against the increase of light net CO_2_ exchange amount. Compared to 4/4 h light/dark cycle, there was no significant change in daily net CO_2_ exchange amount after the light/dark cycle was changed to 12/12 h. During this light/dark cycle switch, the increase of dark net CO_2_ exchange amount was offset by the decrease of light net CO_2_ exchange amount. Compared to the previous 12/12 h light/dark cycle, daily net CO_2_ exchange amount significantly decreased after light/dark cycle was changed back to 12/12 h, due to the decrease of both light net CO_2_ exchange amount and dark net CO_2_ exchange amount.

For *K. daigremontiana*, there were no significant changes in daily net CO_2_ exchange amount during the conversion of different light/dark cycles (Figure 2C). Compared to 12/12 h light/dark cycle, the daily net CO_2_ exchange amount did not change significantly after the light/dark cycle was changed from 12h/12 h to 4/4 h. During this light/dark cycle switch, the increase of light net CO_2_ exchange amount was almost equivalent to the decrease of dark net CO_2_ exchange amount. Compared to the previous 12/12 h light/dark cycle there was no significant change in daily net CO_2_ exchange amount when light/dark cycle was changed back to 12/12 h, due to no significant change in light and dark net CO_2_ exchange amount.

### Dark Net CO_2_ Exchange Percentage

The dark net CO_2_ exchange percentage of *D. officinale*, *D. primulinum*, and *K. daigremontiana* showed an increasing trend during 12/12 h light/dark cycle (Table 1). When the light/dark cycle was changed from 12/12 h to 4/4 h and then back to 12/12 h, the dark net CO_2_ exchange percentage of *D. officinale* and *D. primulinum* changed from a positive value to a negative value and then back to a positive value. Compared to that of the original 12/12 h light/dark cycle, the positive value was significantly decreased for these two *Dendrobium* species after the light/dark cycle was changed back to 12/12 h light/dark cycle from 4/4 h. The dark net CO_2_ exchange percentage of *K. daigremontiana* always remained positive and showed a trend of decreasing first and then increasing during the different light/dark cycles conversion. For *K. daigremontiana*, there was no significant difference in the dark net CO_2_ percentage of 12/12 h light/dark cycle before (85.5%) and after (91.3%) 4/4 h light/dark cycle.

**Table 1:** Effect of light/dark cycle on dark net CO_2_ exchange percentage of *D. officinale*, *D. primulinum*, and *K. daigremontiana*.

Light/dark cycle	Dark net CO_2_ exchange percentage (%)		
	*D. officinale*	*D. primulinum*	*K. daigremontiana*
12/12 h	34.0 ± 3.1 d	56.7 ± 0.3 b	85.5 ± 5.9 a
12/12 h → 4/4 h	−16.8 ±2.9 g	−10.5 ± 2.9 f	21.8 ± 6.0 e
4/4 h → 12/12 h	25.8 ±2.4 e	46.7 ± 1.1 c	91.3 ± 1.9 a

Values were means ± standard deviation. Different letters indicate significant differences by Duncan’s multiple range test (p < 0.05; n = 3).

### Stomatal Conductance

**Figure 3:**
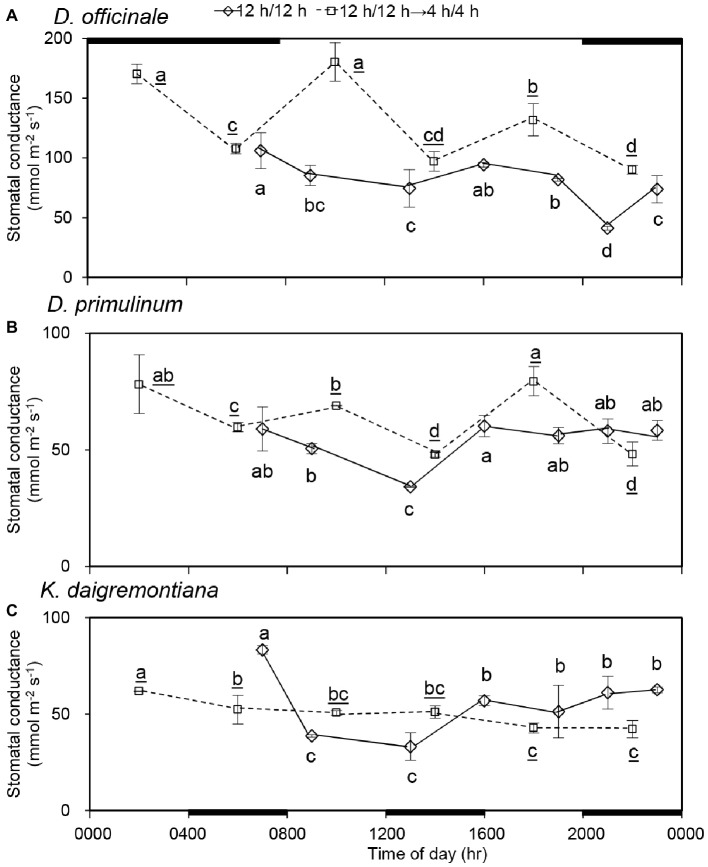
Effect of light/dark cycle on stomatal conductance of *D. officinale* **(A)**, *D. primulinum* **(B)**, and *K. daigremontiana* **(C)**. Data were randomly collected from four plants on day 3 and day 9, respectively (mean ± SD, *n* = 4 for each of these 2 days). The thin black line and thick black line on top indicate light period and dark period of 12/12 h light/dark cycle, respectively. The thin black line and thick black line on the bottom horizontal axis indicate light period and dark period of 4/4 h light/dark cycle respectively. Different letters indicate statistically significant differences by Duncan’s multiple range test (*p* < 0.05). The underlined letters represent differences in 4/4 h light/dark cycle.

Statistically, there was a significant decline in stomatal conductance of *D. officinale* (Figure 3A), *D. primulinum* (Figure 3B), and *K. daigremontiana* (Figure 3C) from 0700 (dark period) to 1300 hours (light period); however, stomatal conductance of all these three species increased significantly from 1300 to 1600 hours during the light period of the 12/12 h light/dark cycle. After light/dark cycle was changed to 4/4 h for 3 days (day 9), the stomatal conductance of *D. officinale* decreased in the dark period and increased in the light period significantly. Although the stomatal behavior of *D. primulinum* exhibited the similar pattern as those of *D. officinale*, the amplitude was much smaller. For *K. daigremontiana*, it showed a gradual decrease in the stomatal conductance from 0200 (light period) to 2200 hours (dark period) 3 days after changing the light/dark cycle from 12/12 h to 4/4 h. Compared to the previous 12/12 h light/dark cycle. After light/dark cycle was changed from 12/12 h to 4/4 h, for *D. officinale* and *D. primulinum*, stomatal conductance of the light period significantly increased, whereas it was not affected for *K. daigremontiana*.

## Discussion

### Effect of Light/Dark Cycle on Net CO_2_ Exchange Pattern and Stomatal Behaviors

Based on the percentage of daily carbon gained by dark fixation, different extent of CAM plants could be easily distinguished (Winter and Holtum, 2002; Zhang et al., 2014; Winter, 2019). Therefore, *D. officinale* was identified as a C3-CAM plant, whereas *D. primulinum* was a CAM plant between the C3-CAM and full CAM. After the light/dark cycle was changed from 12/12 h to 4/4 h for 3 days, net CO_2_ exchange pattern of *D. officinale* and *D. primulinum* switched from CAM defined by Osmond (1978) to C3-like (net CO_2_ uptake in the light and net CO_2_ release in the dark, Figure 1). Stomatal behavior of these two *Dendrobium* species also switched from the CAM pattern to the C3-like pattern (increasing in light period and decreasing in dark period, Figure 3). Tallman (2004) suggested that in phase III of CAM, the photosynthesis of guard cells can obtain a large amount of CO_2_ from the mesophyll and build a strong sink for NADPH, thereby inhibiting the degradation of endogenous ABA in guard cells and promoting stomatal closure (Lind et al., 2015). Therefore, it can be speculated that when the dark CO_2_ absorption decreases to a certain extent, the mesophyll cells will not have enough CO_2_ supply to the guard cells in the light period, so as not to inhibit the opening of the stomata. Some studies related to facultative CAM plants have concluded that light regulates stomatal conductance of these plants only when they are in C3 pattern (Lee and Assmann, 1992; Tallman et al., 1997). Our results were consistent with these previous studies. Net CO_2_ uptake in dark period generally existed in *K. daigremontiana* regardless of light/dark cycle (Figure 2C). However, there were no significant changes in the stomatal conductance during both dark and light periods from 0600 to 2200 hours after changing the light/dark cycle from 12/12 h to 4/4 h (Figure 3). Dark CO_2_ fixation was almost exclusively catalyzed by PEPC in CAM species. The allosteric performance of PEPC was regulated posttranslationally by a circadian clock controlled protein kinase called phosphoenolpyruvate carboxylase kinase (PPCK) (Nimmo et al., 1984, 1986, 1987; Hartwell et al., 1999; Owen and Griffiths, 2013; Boxall et al., 2017; Ohara and Satake, 2017). The activity of PPCK of *Bryophyllum fedtschenkoi* reach appeared several hours after the onset of darkness (Carter et al., 1991). It was possible that when the dark period was too short, the activities of PPCK might not be high enough, which might inhibit the dark net CO_2_ fixation in *D. officinale*, *D. primulinum*, and *K. daigremontiana* to different extents (Figure 2). However, this study did not measure the activity of PPCK of these three species during the conversion of different light/dark cycles, which needs to be studied further in the future.

After the light/dark cycle was changed back to 12/12 h light/dark cycle, dark net CO_2_ exchange percentage of two *Dendrobium* species decreased significantly compared to the original 12/12 h light/dark cycle, but that of *K. daigremontiana* did not (Table 1). However, it did not imply that the 4/4 h light/dark cycle induction suppressed the CAM activity of *D. officinale*. Compared to the original 12/12 h light/dark cycle, dark net CO_2_ exchange amount increased significantly for *D. officinale* after the light/dark cycle was changed back to 12/12 h (Figure 2A).

### CO_2_ Absorption and Stomatal Behaviors

After the light/dark cycle was changed from 12/12 h to 4/4 h, daily net CO_2_ exchange amount increased by 47% for *D. officinale* (Figure 2A). It might be due to the fact that sharp increased stomatal conductance (Figure 3) were favorable to the diffusion of CO_2_ into the leaf, eventually promoting the CO_2_ absorption during the light period of 4/4 h light/dark cycle. After the light/dark cycle was changed from 4/4 h back to 12/12 h, daily net CO_2_ exchange amount of *D. officinale* reached a higher value compared to 4/4 h light/dark cycle (Figure 2A) due to a little down regulation of C3 activity (represented by light net CO_2_ exchange amount) and large up regulation of CAM activity (represented by dark net CO_2_ exchange amount). However, for *D. primulinum*, the daily net CO_2_ exchange amount decreased by 38% after the light/dark cycle was changed from 12/12 h to 4/4 h (Figure 2B), it was possibly due to the fact that its stomatal conductance increased much less than that of *D. officinale* limited the diffusion of CO_2_ into the leaf (Evans and Loreto, 2000), eventually inhibiting the substantial increase of CO_2_ absorption during the light period of 4/4 h light/dark cycle. The contrary responses of daily net CO_2_ exchange amount of *D. officinale* and *D. primulinum* on 4/4 h light/dark cycle may also be related to their different mesophyll conductance (*g*_m_). A strong correlation has been found between *g*_m_ and photosynthetic capacity in two species of Orchidaceae, and *g*_m_ was mainly determined by surface area of mesophyll cells, chloroplasts exposed to intercellular airspace per unit of leaf area and cell wall thickness (Yang et al., 2018). Reduced intercellular air space (IAS) and reduced surface of mesophyll exposed to IAS (*L*_mes_/area) were positively related to CAM function, and negatively related to C3 function (Nelson and Sage, 2008). Structure features of leaf may affect CO_2_ concentration in chloroplast stroma (Terashima et al., 2011). It was reported that increased O_2_/CO_2_ eventually increased photorespiration. Photorespiration had an especially high demand for energy (Osmond and Grace, 1995; Heber et al., 2001; Heber, 2002). After the light/dark cycle changed from 12/12 h to 4/4 h, the daily net CO_2_ exchange amount of *D. primulinum* decreased significantly, which suggested that *D. primulinum* might subject to severe photorespiration during the light period of 4/4 h light/dark cycle. Therefore, the changes of daily net CO_2_ exchange amount of *D. officinale* and *D. primulinum* were reversed after the photosynthetic pathway was switched from CAM to C3. This may be related to stomatal conductivity, *g*_m_, cell wall conductance, cytosol conductance, stromal conductance, etc., which require further study to clarify the relationship between leaf functional structure and photosynthesis of *Dendrobium* plants.

## Conclusions

The responses of gas exchange and stomatal movement of two *Dendrobium* species to different light/dark cycles conversion were evaluated in this study. The net CO_2_ exchange pattern and stomatal behavior of *D. officinale* and *D. primulinum* could be switched from CAM to C3-like by changing the light/dark cycle from 12/12 h to 4/4 h. However, this switching was not completely reversible as the dark, light, and daily net CO_2_ exchange amount of *D. officinale* were significantly increased after the light/dark cycle was changed from 4/4 h to 12/12 h compared to the original 12/12 h light/dark cycle. The responses of *D. primulinum* to different light/dark cycle conversion were opposite from those of *D. officinale*. The net CO_2_ uptake during the dark period was always present in *K. daigremontiana* regardless of light/dark cycle.

Daily net CO_2_ exchange amount of *D. officinale* was enhanced by changing the light/dark cycle from 12/12 h to 4/4 h, but that of *D. primulinum* was inhibited. The daily net CO_2_ exchange amount of *K. daigremontiana* was not affected by different light/dark cycles conversion.

## Author Contributions

YC carried out the experiments and wrote the manuscript. DH and RG designed the experiments and participated in the statistical data evaluation. JH and GN reviewed and edited the manuscript.

### Conflict of Interest Statement

The authors declare that the research was conducted in the absence of any commercial or financial relationships that could be construed as a potential conflict of interest.
